# FALS-YOLO: An Efficient and Lightweight Method for Automatic Brain Tumor Detection and Segmentation

**DOI:** 10.3390/s25195993

**Published:** 2025-09-28

**Authors:** Liyan Sun, Linxuan Zheng, Yi Xin

**Affiliations:** College of Computer Science and Technology, Changchun University, No. 6543, Satellite Road, Changchun 130022, China; sunly@ccu.edu.cn (L.S.); 231502531@mails.ccu.edu.cn (Y.X.)

**Keywords:** brain tumor, object detection, image segmentation, lightweight, multi-scale features

## Abstract

Brain tumors are highly malignant diseases that severely threaten the nervous system and patients’ lives. MRI is a core technology for brain tumor diagnosis and treatment due to its high resolution and non-invasiveness. However, existing YOLO-based models face challenges in brain tumor MRI image detection and segmentation, such as insufficient multi-scale feature extraction and high computational resource consumption. This paper proposes an improved lightweight brain tumor detection and instance segmentation model named FALS-YOLO, based on YOLOv8n-Seg and integrating three key modules: FLRDown, AdaSimAM, and LSCSHN. FLRDown enhances multi-scale tumor perception, AdaSimAM suppresses noise and improves feature fusion, and LSCSHN achieves high-precision segmentation with reduced parameters and computational burden. Experiments on the tumor-otak dataset show that FALS-YOLO achieves Precision (B) of 0.892, Recall (B) of 0.858, mAP@0.5 (B) of 0.912 in detection, and Precision (M) of 0.899, Recall (M) of 0.863, mAP@0.5 (M) of 0.917 in segmentation, outperforming YOLOv5n-Seg, YOLOv8n-Seg, YOLOv9s-Seg, YOLOv10n-Seg and YOLOv11n-Seg. Compared with YOLOv8n-Seg, FALS-YOLO reduces parameters by 31.95%, computational amount by 20.00%, and model size by 32.31%. It provides an efficient, accurate and practical solution for the automatic detection and instance segmentation of brain tumors in resource-limited environments.

## 1. Introduction

**Figure 1:**
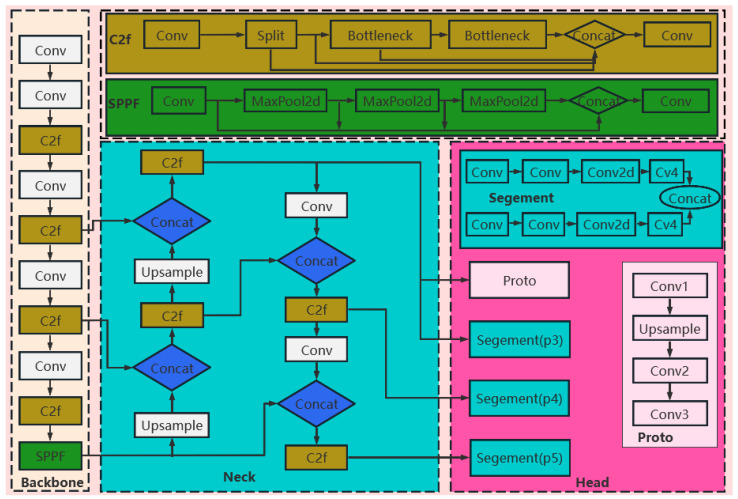
Structure diagram of the YOLOv8n-Seg model.

Brain tumors are diseases that originate from abnormal cell clusters within the skull, characterized by high malignancy and rapid growth, posing great harm to the function of the nervous system. According to the statistics of the World Health Organization (WHO), the annual incidence rate of brain tumors worldwide is about 6–8 per 100,000 people, that is, on average, about 6 to 8 people out of every 100,000 are newly diagnosed with brain tumors each year [1]. Not only is the incidence rate high, but the number of deaths caused by brain tumors each year is also considerable. Even in countries and regions with relatively developed medical conditions, this trend is still evident. Based on the data from the National Cancer Institute (NCI) of the United States, it is estimated that in 2024, about 18,760 people in the United States die from brain and central nervous system tumors [2]. Magnetic resonance imaging (MRI), as a typical non-invasive imaging technology, can generate high-quality, non-invasive and skull-artifact-free brain images, providing comprehensive and accurate information for the diagnosis and treatment of brain tumors, and has become the main technical means for the diagnosis and treatment of brain tumors [3]. MRI has a high soft tissue contrast, and the imaging effects of different modality images in different regions of brain tumors vary, which can provide doctors with a wealth of diagnostic basis. Determining the location, maximum diameter, volume and quantity of the lesion tissue is the basis for formulating the diagnosis and treatment plan, and the accurate localization and segmentation of brain tumors are the prerequisites for making these judgments. Despite the significant role of medical image analysis in brain tumor diagnosis, traditional image interpretation methods mainly rely on manual operations, which are characterized by subjectivity, low efficiency, time-consuming nature, and limited accuracy. At present, clinical diagnosis still primarily depends on manual annotation, not only consuming a great deal of human resources but also showing significant differences in annotation results among different doctors. Moreover, due to the complex anatomical structures in MRI images and the diverse shapes of brain tumors, high professional capabilities are required of doctors. Therefore, it is of great theoretical value and practical application prospect to study a method that can achieve automatic detection and segmentation of brain tumors. With the rapid development of artificial intelligence (AI) technology in the field of computer vision, automated medical image analysis has shown great potential. By introducing AI technology, not only can the diagnostic efficiency be significantly improved, but the consistency and accuracy of diagnosis can also be enhanced, which is of great significance for clinical auxiliary diagnosis. In recent years, deep learning algorithms such as convolutional neural networks (CNN) [4], object detection frameworks (such as the YOLO series) [5,6,7,8,9,10,11,12,13,14,15], and image segmentation models (such as U-Net, etc.) [16,17,18] have been widely applied in the field of medical image analysis, becoming important tools for improving diagnostic accuracy and processing efficiency. Among them, object detection models based on the YOLO (You Only Look Once) architecture—especially YOLOv8—offer not only efficient detection performance but also powerful instance segmentation capabilities. These models are increasingly attracting attention in the field of medical image analysis and provide an innovative solution for the automatic recognition of brain tumors. YOLOv8 adopts a unified multi-task learning architecture, in which the YOLOv8–Seg (Figure 1) variant can simultaneously achieve object detection, instance segmentation and image classification functions, greatly expanding its scope of application in diverse visual tasks. In terms of model structure design, YOLOv8 introduces the Decoupled Head, which separates the classification and bounding box regression tasks, thereby effectively improving the recognition accuracy of small objects and edge areas. YOLOv8 employs CSPDarknet as its backbone, enhanced with the SE module and PANet to boost feature extraction and multi-scale fusion. It adopts an Anchor-Free design, eliminating manual anchor tuning and improving adaptability to objects of varying sizes and shapes—particularly beneficial for accurate boundary segmentation. For segmentation, it combines Dice Loss and BCE Loss to address class imbalance and better capture boundary details. By integrating advanced data-augmentation techniques such as Mosaic and MixUp, as well as optimization designs for inference speed, YOLOv8 has real-time processing capabilities at the millisecond level while maintaining high accuracy [19]. Therefore, YOLOv8 has a wide range of application prospects in many fields such as autonomous driving, industrial quality inspection, intelligent security and medical image analysis [20]. Compared with semantic segmentation methods such as U-Net, YOLOv8-Seg performs instance-level segmentation, enabling not only discrimination among lesion categories but also precise localization and delineation of multiple individual instances within the same class—thereby supporting clinical quantification and treatment assessment. Its high inference efficiency and strong cross-dataset generalization significantly enhance the practicality of medical image segmentation. Although YOLOv8-Seg demonstrates promising performance, there remains room for further optimization. For example, the feature extraction module can be improved to more accurately capture the edge details and shape changes in the tumor; the introduction of attention mechanisms can effectively deal with problems such as fuzzy tumor contours, complex deformations, and background interference; at the same time, appropriately simplifying the network structure to reduce the number of parameters and computational overhead can help improve its deployment efficiency in resource-constrained medical environments. The contributions of this study are as follows:

1. Novel Downsampling Module FLRDown: This study designs a novel downsampling module FLRDown that integrates low-rank convolution and Fourier transform. While reducing spatial resolution, this module can more effectively preserve the low-frequency global information of images, enhance the model’s receptive field and feature expression ability, and at the same time lower computational complexity, thus providing stronger support for the recognition of multi-scale targets in medical images.
2. Adaptive Simple Parameter-Free Attention Module AdaSimAM: Based on the SimAM attention mechanism, this study proposes a new attention module named AdaSimAM. AdaSimAM effectively reduces the interference of high-frequency noise on feature expression and enhances the feature response of local areas. Moreover, this module introduces an adaptive parameter-adjustment mechanism, enabling the model to more flexibly adapt to the needs of multi-scale features in visual tasks such as object detection and semantic segmentation.
3. Lightweight Segmentation Convolutional Segmentation Head Network LSCSHN: In response to the issue of large parameter quantity and high resource consumption in the original YOLO series models, this invention designs the LSCSHN segmentation head network. It optimizes the head network structure and reduces redundant parameters. LSCSHN maintains high-precision output while significantly lowering the model’s computational load and enhancing its deployment capability in resource-constrained environments.

## 2. Related Work

R. Ranjbarzadeh et al. proposed a cascade CNN with a distance-wise attention mechanism to improve the accuracy and efficiency of brain tumor segmentation in MRI images [21]. 

The team of Naira Elazab constructed a multi-category brain tumor grading system [22], combining the strengths of YOLOv5 and ResNet50, using YOLOv5 for region localization and ResNet50 for deep feature extraction, and introducing an XGBoost classifier to enhance classification performance under high-dimensional features, achieving more accurate tumor type identification. Lin Y., Lin J., and Jiang Z. proposed an improved YOLOv8-DEC model [23], which effectively enhanced the accuracy of brain tumor detection by integrating CARAFE, EMA, and DSConv modules. S. Priyadharshini et al. proposed a comparative framework based on YOLOv9–v11 for brain tumor detection and segmentation in MRI, incorporating preprocessing and segmentation-aligned pipelines. YOLOv11 achieves the best performance, demonstrating high efficiency and potential for real-time applications [24]. M. S. Mithun et al. proposed a brain tumor detection and classification method based on the YOLO NAS deep learning model, targeting four categories in MRI images: pituitary tumor, meningioma, glioma, and no tumor [25]. The method first employs Hybrid Anisotropic Diffusion Filtering (HADF) to remove image noise and then uses the En–DeNet network—comprising a U-Net-based encoder and a pre-trained EfficientNet decoder—to automatically segment tumor regions. Subsequently, YOLO NAS combined with EfficientDet and the DETR3 Transformer is applied to extract multi-scale features, refine detection results, and leverage an attention mechanism to suppress background noise, thereby improving classification accuracy. Aruna Chen et al. proposed YOLO-NeuroBoost, an improved YOLOv8-based model for brain tumor detection in MRI, incorporating KernelWarehouse, CBAM, and Inner-GIoU. It achieves high accuracy and robustness, especially for small tumors, and enhances interpretability via Grad-CAM [26]. Seyed Mohammad Hossein Hashemi et al. proposed a two-stage deep-learning framework that combines YOLOv8 and DeiT [27] for brain tumor detection and classification. This method uses a lightweight YOLOv8n model for fast localization in the first stage and DeiT and Vision Transformer (ViT) modules for feature enhancement and classification decision-making in the second stage, enhancing the overall diagnostic effect. Bonagiri Dinesh Kumar Reddy et al. also explored the application of YOLOv8 in MRI images [28], pointing out that it performs well in both object detection and image segmentation tasks, especially YOLOv8-Seg. The ASF-YOLO proposed by Ming Kang et al. [29] introduces several modules such as attention-based scale-sequence fusion (SSFF), triple feature encoding (TFE), and channel-and position-attention mechanisms (CPAM) for cell instance segmentation tasks, significantly improving the segmentation effect of small objects and also providing new ideas for the fine segmentation of brain tumor MRI images. Maram Fahaad Almufareh et al. developed a YOLO-based method specifically for the detection and segmentation of three types of tumors: meningioma, glioma, and pituitary tumor [30]. This method combines the bounding box and mask prediction functions of YOLOv5 and YOLOv7 and is further enhanced by advanced preprocessing techniques to improve model performance. Pankaj Kasar et al. proposed two CNN methods based on U-Net and SegNet for automatic tumor segmentation in MRI images [31]. U-Net, with its symmetric encoder–decoder structure and skip connections, performs well in high-resolution image processing. In contrast, SegNet, which uses max-pooling indices for upsampling, improves computational efficiency but is slightly inferior to U-Net in segmentation accuracy. S. Karthikeyan et al. used Roboflow for data annotation and training and implemented precise detection of brain tumors in MRI images based on YOLOv8 [32], emphasizing the potential of customized YOLOv8 in medical image analysis. Nur Iriawan et al. proposed the YOLO-UNet architecture [33], which combines the object detection capability of YOLO with the segmentation ability of UNet. The YOLO first locates the tumor region, and then the UNet performs edge segmentation. This method is particularly suitable for tumors with complex or variable shapes, effectively improving the accuracy of detection and segmentation. Zafar W. and Husnain G. et al. further improved this idea and proposed Enhanced TumorNet [34], which integrates the YOLOv8s and U-Net architectures. First, the U-Net performs precise segmentation of the tumor region, followed by efficient detection and classification using YOLOv8s. Experiments have shown that this method performs excellently in both segmentation and recognition tasks. Finally, Abdulkadir Karaci et al. proposed the YoDenBi-NET model [35], which combines YOLO, DenseNet, and Bi-LSTM technologies to form a three-stage processing workflow: YOLO is responsible for object detection, DenseNet extracts image features, and Bi-LSTM completes the final serialized classification task, demonstrating good adaptability and scalability in brain tumor classification tasks. Y. Lyu and X. Tian proposed a hybrid Transformer U-Net model, termed MWG-UNet++, for brain tumor segmentation in MRI scans [36]. The model integrates the U-Net architecture with enhanced Transformer layers and incorporates Wasserstein generative adversarial networks (WGAN) for data augmentation to improve segmentation accuracy. Deependra Rastogi et al. proposed a deep learning framework based on MRI scans for precise brain tumor segmentation and survival prediction in glioma patients [37]. The study employed a 2D volumetric convolutional neural network architecture to achieve robust and reliable tumor segmentation and incorporated a majority voting strategy to significantly reduce model bias and enhance performance. Asadullah Shaikh et al. proposed an advanced stacking ensemble learning (SEL) framework, employing a stacked DenseNet201 as the meta-model and integrating six diverse base learners—MobileNet-v3, 3D-CNN, VGG-16, VGG-19, ResNet50, and AlexNet—to enhance brain tumor detection and segmentation from MRI scans [38]. The study highlights the effectiveness of ensemble-based deep learning approaches in improving the robustness and accuracy of early brain tumor diagnosis and treatment planning. Ganesh et al. proposed a brain tumor segmentation and detection method based on a convolutional neural network (CNN) and the VGG16 model [39]. In this study, MRI brain images were processed using an improved texture detection algorithm—Spatial Gray-Level Dependence Matrix (SGLD)—and integrated image processing techniques were employed to achieve automatic tumor identification. Furthermore, the study demonstrated how the segmentation problem could be transformed into a classification task by distinguishing normal and abnormal pixels using features such as intensity and texture.

Although the existing models based on U-net or those combined with the Transformer variant models have made significant progress in the efficiency and accuracy of semantic segmentation for brain tumors in brain MRI images, the brain tumor detection and instance segmentation based on YOLO still have several limitations, mainly manifested in the following aspects: First, these models have limited ability to extract features from multi-scale tumor regions and cannot fully capture key details, which often leads to a decrease in segmentation accuracy. Second, the model structures are relatively complex and consume a large number of computational resources. This not only increases training time but also restricts their deployment and application in medical environments with limited resources. Finally, when facing the diversity of brain tumor shapes, sizes, and locations, existing models are insufficient in terms of adaptability and robustness and cannot stably and accurately perform segmentation tasks in complex scenarios. Therefore, this study adopts YOLOv8-Seg as the baseline framework and introduces targeted improvements, focusing on optimizing the network architecture, enhancing multi-scale feature extraction, and incorporating advanced feature fusion mechanisms. These enhancements effectively reduce model complexity while maintaining high detection and segmentation accuracy, thereby improving the robustness and generalization capability of brain tumor detection and instance segmentation.

## 3. Materials and Methods

**Figure 2:**
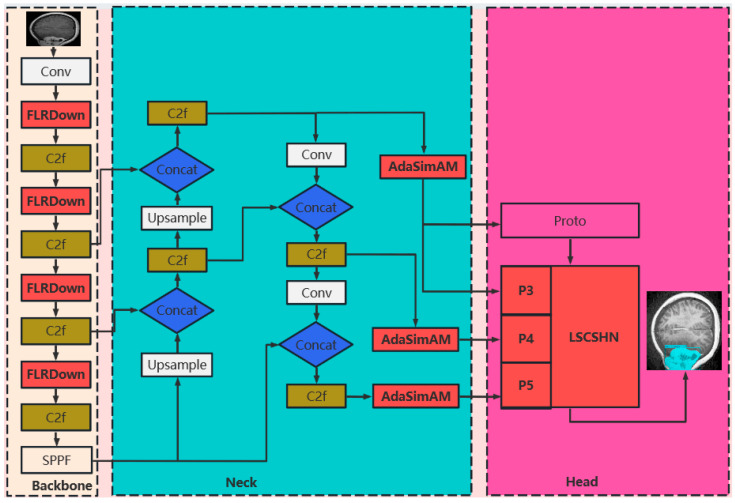
Structure diagram of the FALS-YOLO model.

In this study, we present FALS-YOLO, a refined YOLOv8-Seg architecture tailored for simultaneous brain tumor detection and instance segmentation, as shown in Figure 2, which aims to enhance its performance in object detection and instance segmentation of brain tumor MRI images by introducing three key modules. First, we replace the original downsampling structure in the backbone network with the novel downsampling module FLRDown, which significantly enhances the model’s perception of multi-scale targets and ensures that tumors of different sizes can be effectively identified and classified. Second, the adaptive simple parameter-free attention module AdaSimAM is embedded in the neck network. This module can dynamically adjust the importance of each feature channel without introducing additional parameters, thereby effectively suppressing noise interference and enhancing the fusion effect of multi-scale features, further improving the robustness and accuracy of the model. Finally, the lightweight segmentation head network LSCSHN optimizes the original segmentation head. While greatly reducing computational overhead, it maintains high-quality segmentation accuracy. Building upon the efficient and accurate characteristics of the YOLO series, FALS-YOLO significantly enhances feature representation capability and substantially reduces model complexity through three core improvements. Experimental results demonstrate that the model achieves outstanding performance in brain tumor detection and segmentation tasks, offering a novel, high-precision yet lightweight solution for instance-level brain tumor segmentation.

### 3.1. FLRDown: An Efficient Downsampling Module Combining Low-Rank Convolution and Fourier Transform

**Figure 3:**
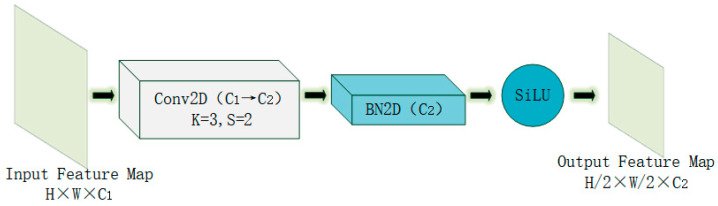
The structure diagram of the down-sampling module Conv in the YOLOv8-Seg backbone network.

In the original YOLOv8-Seg backbone network, downsampling is implemented using a conventional convolution operation module (Conv as shown in Figure 3). For example, a convolution operation with a kernel size of 3 × 3 and a stride of 2 is used to downsample the feature map, halving its size each time. For instance, when the input feature map size is 256 × 256, after one convolution-based downsampling operation, the feature map will be reduced to 128 × 128. Although this conventional convolution module simplifies the operation process to some extent, its feature extraction capability is relatively insufficient, which can easily lead to the loss of key features and thus affect the overall accuracy of the model. In addition, this module has a large computational load and performs poorly in resource-constrained environments. To address these issues, this study proposes a novel downsampling module named FLRDown to replace the conventional convolution operation module in the YOLOv8n-Seg backbone network. The FLRDown module integrates low-rank convolution and Fourier transform, which not only effectively reduces the spatial resolution but also more fully retains the low-frequency global information of the image. This design significantly enhances the model’s receptive field and feature expression ability while reducing the overall computational complexity, providing stronger technical support for brain tumor recognition tasks.

**Figure 4:**
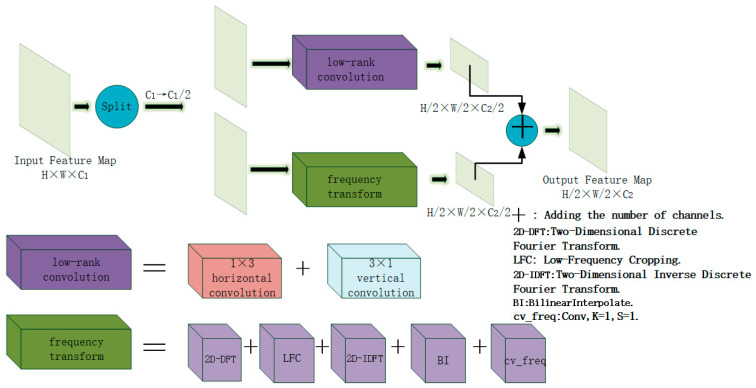
The structure diagram of the down-sampling module FLRDown in the FALS-YOLO backbone network.

The structure of FLRDown is shown in Figure 4. This module combines low-rank convolution and frequency-domain transform to reduce the spatial resolution and channel number of the feature map while maintaining the compactness and effectiveness of the feature representation. The input feature map is first divided into two branches along the channel dimension, with each branch containing C1/2 channels. One of the branches performs a low-rank convolution operation. The traditional 3 × 3 convolution, although capable of extracting rich local features, has a high computational complexity because each pixel needs to consider its eight neighboring pixels. This is especially significant in deep neural networks, where it brings considerable computational overhead. To address this, the module employs a low-rank convolution strategy in the spatial branch, decomposing the standard 3 × 3 convolution into a horizontal 1 × 3 convolution followed by a vertical 3 × 1 convolution. The horizontal convolution is performed first, followed by the vertical convolution (with the 3 × 1 convolution set to a stride of 2). This approach effectively reduces the number of parameters and computations while achieving an equivalent receptive field and completing feature dimensionality reduction and spatial downsampling. It enhances spatial representation capabilities while maintaining a low computational cost, ultimately outputting a feature map of size *H*/2 × *W*/2 × *C*_2_/2. To make up for the deficiency of low-rank convolution in global modeling, this module introduces a frequency-domain branch. The feature maps input to this branch undergo frequency-domain transformation operations to enhance the modeling of long-range dependencies. In the frequency-domain branch, the input feature map is first mapped to the frequency domain via the 2D-DFT (Equation (1)), and then the redundant high-frequency components are removed through the LFC (Equation (2)), retaining only the low-frequency information that is more discriminative for the tumor region. Subsequently, the features are remapped back to the spatial domain via the 2D-IDFT (Equation (3)) and resized through bilinear interpolation (BI) to align with the output of the spatial branch. Finally, the feature representation is further refined using a frequency-domain convolution (cv_freq), resulting in an output feature map of size *H*/2 × *W*/2 × *C*_2_/2. The two branches are then concatenated along the channel dimension to produce the final fused output feature map, which has a size of *H*/2 × *W*/2 × *C*_2_. By integrating the feature extraction capabilities of both the spatial and frequency domains, the FLRDown module enhances the efficiency of feature downsampling while balancing global and local representation abilities. It is particularly well-suited for tasks such as image super-resolution, multi-scale feature extraction, and brain tumor detection and segmentation, where computational efficiency is of utmost importance.

(1) $$F_{\left(u,v\right)}=\sum_{m=0}^{H-1}\sum_{n=0}^{W-1}\times (m,n)e^{-j2\pi (\frac{um}{H}+\frac{vn}{W})}$$

(2) $$F_{low}(u,v)=F[:,:,:u,:v],\;0\leq u<H/2,\;0\leq v<W/2$$

(3) $$X^{\prime }(m,n)=\sum_{u=0}^{H/2}\sum_{v=0}^{W/2}F_{low}(u,v)e^{j2\pi (\frac{um}{H}+\frac{vn}{W})}$$

$F_{\left(u,v\right)}$: The Fourier transform result of the input feature x in the frequency domain.

u,v: Frequency domain coordinates.

m,n: Space coordinates.

j: Imaginary unit.

H,W: The height and width of the feature map.

$F_{low}\left(u,v\right):$ The cropped low-frequency part.

$X^{\prime }(m,n)$: The spatial-domain features recovered from the low-frequency information.

The backbone network of FALS-YOLO utilizes FLRDown as the efficient downsampling module and combines it with the C2f module and the SPPF (Spatial Pyramid Pooling Fast) module to successively perform multiple downsampling and feature extraction operations, thereby achieving the acquisition of multi-scale features. Specifically, the network ultimately outputs three sets of feature maps with different resolutions: P3/8 (shallow-level feature map), P4/16 (mid-level feature map), and P5/32 (deep-level feature map), which are used to capture spatial and semantic information at different hierarchical levels in the image. These multi-scale feature maps will then be passed to the neck network to perform multi-scale fusion and detail enhancement operations, laying a solid feature-based foundation for the subsequent brain tumor detection and segmentation tasks.

### 3.2. AdaSimAM—An Adaptive Simple Parameter-Free Attention Module

**Figure 5:**
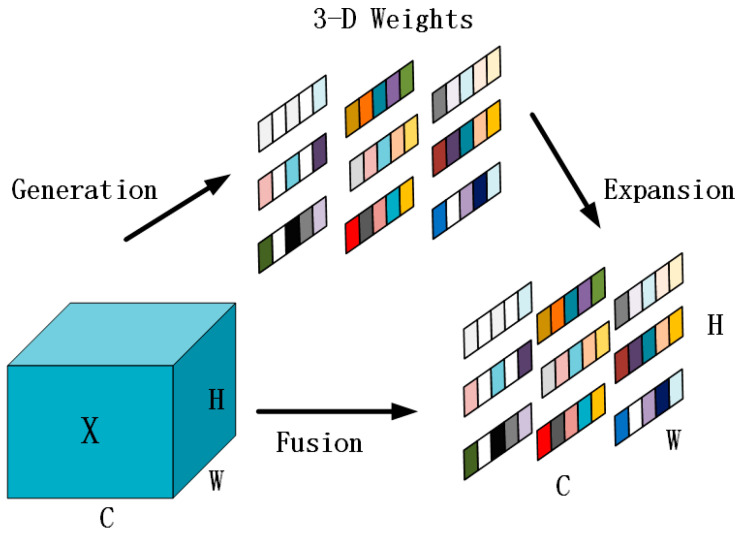
The SimAM module is capable of directly estimating the 3-D weights. In each sub-figure, the same color indicates that the same scalar value is shared for each channel, each spatial location, or each point on the feature.

SimAM (Simple Parameter-Free Attention Module) is a simple and parameter-free attention mechanism that cleverly combines channel attention and spatial attention to generate 3-D weights without adding extra parameters (as shown in Figure 5). It effectively highlights the important features of the feature map and suppresses irrelevant parts, showing great performance in enhancing the feature representation ability of convolutional neural networks [40]. However, SimAM also has some drawbacks. To address these shortcomings, this study proposes the improved AdaSimAM (Adaptive, Simple, Parameter-Free Attention Module) and applies it to the brain tumor segmentation task of the YOLOv8n-Seg model. Specifically, the AdaSimAM attention module is inserted after the outputs of different levels of the neck network to further enhance the feature representation of the feature maps output by different levels of the neck network, thereby improving the overall performance of the model without significantly increasing the number of model parameters and computational complexity.

The shortcomings of SimAM are mainly reflected in the following aspects: First, it is sensitive to noise and lacks an effective mechanism to reduce the impact of noise in the performance. Second, SimAM uses the global mean as the feature processing method, ignoring local feature information, which may perform poorly in tasks with high detail requirements, such as object detection and semantic segmentation. Finally, the hyperparameter $e_{\lambda }$ in SimAM is used to adjust the sensitivity of the attention mechanism, which is input feature map, which may lead to inaccurate attention weights and thus affect model crucial for ensuring the stability and effectiveness of the model training process. In practical applications, it may be necessary to adjust the value of $e_{\lambda }$ appropriately according to specific situations to obtain the best performance. However, in SimAM, $e_{\lambda }$ is fixed and cannot be dynamically adjusted according to the changes in the features of multi-scale image data, which limits its ability to adapt to different feature distributions and reduces the model’s generalization ability. To address the shortcomings of SimAM, AdaSimAM proposes several key improvements. First, smoothing is used to reduce noise interference. By applying average pooling to the input feature map before calculating the local mean, the impact of noise is effectively reduced. Second, the local window mean is used instead of the global mean, with the global mean only used when the feature map size is small enough or the window size is 1, thus better capturing local feature information in the image. Finally, AdaSimAM introduces an adaptive hyperparameter $Adaptive\_e_{\lambda }$, which is dynamically adjusted according to the standard deviation of the input feature map to enhance the model’s adaptability to different data distributions. These improvements enable the model to perform better in handling noise, local features, and hyperparameter adjustment.

The workflow of the improved AdaSimAM attention module in the proposed model of this invention is as follows: The three-level feature maps output from the multi-scale feature fusion of the neck network are, respectively, input into the AdaSimAM attention module. First, average pooling is used to smooth the input feature maps (Equation (4)), effectively reducing the interference of high-frequency noise on feature expression and thereby enhancing the stability and accuracy of attention calculation. Subsequently, local-window average pooling is employed to extract statistical features of local regions (Equations (5) and (6)), replacing the traditional global-mean operation to enhance the model’s ability to focus on detail areas and better adapt to the sensitivity requirements for local features in tasks such as object detection and semantic segmentation. The difference between the smoothed feature map and its local mean is used as the core information to calculate the attention response at each spatial location. The relative importance is measured through normalization to generate channel-level attention weight maps. The hyperparameter $Adaptive\_e_{\lambda }$ (Equations (7)–(9)) is dynamically adjusted according to the standard deviation of the input feature map in the spatial dimension, enabling the attention mechanism to have stronger adaptability to input features of different scales and thus improving the model’s generalization performance. Finally, the calculated attention weights are element-wise multiplied with the original input feature maps to obtain the weighted output feature maps P3-s, P4-m, and P5-l, which provide more discriminative feature representations for the subsequent head network.

(4) $$x^{\prime }=\mathrm{AvgPool}(x,\mathrm{kernel}\text{\_}\mathrm{size}=\mathrm{smooth}\text{\_}\mathrm{kernel}\text{\_}\mathrm{size},\mathrm{stride}=1,\;\mathrm{padding}=\frac{\mathrm{smooth}\text{\_}\mathrm{kernel}\text{\_}\mathrm{size}}{2})$$

(5) $$\mathrm{local}\text{\_}\mathrm{mean}\;=\;\mathrm{AvgPool}(x^{\prime },\mathrm{kernel}\text{\_}\mathrm{size}=\mathrm{local}\text{\_}\mathrm{window}\text{\_}\mathrm{size},\mathrm{stride}=1,\;\mathrm{padding}=\frac{\mathrm{local}\text{\_}\mathrm{window}\text{\_}\mathrm{size}}{2})$$

(6) $$\mathrm{local}\text{\_}\mathrm{mean}=\frac{1}{H\times W}\sum_{i=1}^{H}\sum_{j=1}^{W}x^{\prime }\left(i,j\right)$$

The hyperparameter is adaptively adjusted $Adaptive\_e_{\lambda }$, and the formula is as follows:

(7) $$\mathrm{Adaptive}\_e_{\lambda }=\sigma_{x}\times \alpha$$

 α: The scaling factor, which is empirically taken as a small constant value, such as 0.01.

$\sigma_{x}:$ The standard deviation of the input feature map *x* in the spatial dimension, calculated separately for each channel (Equation (8)).

$x_{c,i,j}$: The pixel value at position (i,j) in channel c.

$\mu_{c}$: The mean value of channel c (Equation (9)).

(8) $$\sigma_{x}=\sqrt{\frac{1}{H\times W}\sum_{i=1}^{H}\sum_{j=1}^{W}(x_{c,i,j}-\mu_{c}{)}^{2}}$$

(9) $$\mu_{c}=\frac{1}{H\times W}\sum_{i,j}x_{c,i,j}$$

### 3.3. LSCSHN: A Lightweight Shared Convolutional Segmentation Head Network

In the original YOLOv8n-Seg model, the head network consists of three segmentation heads, which is the key part for implementing the final detection and segmentation decisions. It is responsible for generating accurate segmentation results by utilizing the multi-scale feature maps (P3-s, P4-m, and P5-l) from the neck network. The segmentation head structure inherits the core design of the YOLOv8 detection head, retaining the detection function and expanding the specialized modules for segmentation tasks. Considering the sensitivity of instance segmentation to target size variations, the head network needs to have stronger multi-scale perception capabilities to effectively model targets of different sizes. However, to enhance multi-scale feature extraction capabilities, more convolution operations and complex feature transformation modules are usually required. This will significantly increase the model’s number of parameters and computational overhead, thereby affecting inference efficiency and deployment flexibility.

**Figure 6:**
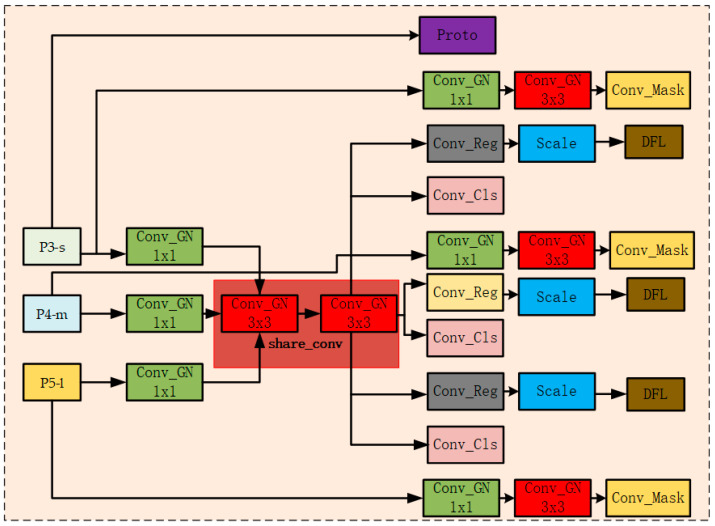
Structural diagram of LSCSHN.

To address the above issues, this paper proposes a lightweight shared convolutional segmentation head structure, named LSCSHN (Lightweight Shared Convolutional Segmentation Head Network, as shown in Figure 6). Compared with the original Segment segmentation head, LSCSHN has made several optimizations in structural design. First, LSCSHN introduces a channel-shared convolution structure, that is, using a shared convolution module (share_conv) for feature maps of different scales. This effectively reduces the number of redundant modules and significantly lowers the number of parameters and memory usage. Second, during the feature transformation process, the Conv_GN module based on Group Normalization is used to replace the traditional Batch Normalization, which improves stability and generalization ability in small-batch training scenarios. In addition, the Scale module is introduced in the regression branch to adjust the scale of regression outputs. This helps control the numerical range of regression values, enhances training stability, and achieves more precise bounding box predictions. During the inference stage, LSCSHN maintains the same post-processing logic as the original Segment to ensure compatibility of output formats. In mask prediction, LSCSHN also uses a shared structure to generate mask coefficients, retaining the original prototype mask generation module (Proto) and mask coefficient prediction module (Conv_mask) to ensure the performance of instance segmentation. Overall, LSCSHN significantly improves the model’s inference efficiency and deployment friendliness while maintaining segmentation accuracy through shared structural design and lightweight module replacement. It is particularly suitable for edge-device scenarios with limited computational resources.

The processing workflow of the LSCSHN model is divided into three stages. First, in the feature-enhancement stage, the multi-scale feature maps (P3-s, P4-m, and P5-l) from the neck network are channel-compressed by a 1 × 1 lightweight convolution module (Conv_GN) and then input into the shared convolution module (share_conv). Here, two-layer cascaded 3 × 3 convolutions (Conv_GN) are employed to enhance the features. Next, in the object-detection stage, the enhanced features at each scale are separately passed through independent regression heads (Conv_Reg) and classification heads (Conv_Cls) to output bounding-box offsets and class probabilities. The bounding-box regression loss (Bbox.Loss, Equation (11)) and classification loss (Cls.Loss, Equation (12)) are calculated. After processing by the scale-adjustment module (Scale, Equation (10)), the final bounding-box coordinates and classification results are decoded using DFL (Distribution Focal Loss), and the distributed focal loss (Dfl.Loss, Equation (13)) is calculated. Finally, in the instance segmentation stage, the shallowest-level feature map (P3-s) is used for the prototype-mask generation module (Proto) to extract a set of prototype masks shared across the entire image. Meanwhile, the feature maps at each scale are processed through a lightweight mask branch (including 1 × 1 and 3 × 3 Conv_GN) to generate corresponding mask coefficients. These coefficients are linearly combined with the prototype masks by Conv_mask to reconstruct high-quality instance segmentation masks for each target, and the mask-segmentation loss (Seg.Loss, Equations (14)–(16)) is calculated to generate the final instance segmentation results.

(10) $$\hat{t}=\theta \cdot t$$

t: The raw predictions output by the regression head (i.e., the bounding box offset predictions for each pixel).

θ: A learnable scalar parameter, initialized to 1.0 and optimized through gradient descent during training.

$\hat{t}$: The scaled prediction result, which is fed into the DFL decoder as the input for distance distribution.

(11) $$Bbox.Loss=\sum_{i=1}^{N}(x_{i}-\bar{x}{)}^{2}$$

$x_{i}$: The coordinates of the ground-truth bounding box.

$\overset{\_}{x}$: The coordinates of the predicted bounding box.

(12) $$Cls.Loss=-\sum_{c=1}^{L}y_{o,c}\log(p_{o,c})$$

y(o,c): An indicator that is 1 if sample o belongs to class c and 0 otherwise.

p(o,c): The probability that the model predicts sample o belongs to class *c*.

L: The number of classes.

(13) $$Dfl.Loss=\sum_{i=1}^{reg\_max\sum }-\log(p_{i})\cdot q_{i}$$

$p_{i}$, $q_{i}$: The predicted probability distribution and the true distribution.

reg_max: Control the granularity of discretized regression and determine the range of distribution prediction.

The segmentation mask loss Seg.Loss is calculated using a combination of binary cross-entropy loss (*BCE*) and Dice loss (Dice.Loss).

(14) $$BCE=-\frac{1}{N}\sum_{i=1}^{N}[M_{i}\log({\hat{M}}_{i})+(1-M_{i}\log(1-{\hat{M}}_{i})]$$

(15) $$Dice=1-\frac{2{\sum_{i}\hat{M}}_{i}M_{i}+\varepsilon }{\sum_{i}{\hat{M}}_{i}+\sum_{i}M_{i}+\varepsilon }$$

(16) $$Seg.Loss=BCE(\hat{M},M)+Dice(\hat{M},M)$$

N: The number of pixels.

$M_{i}$: The true mask pixel value of the i-th pixel.

${\hat{M}}_{i}$: The predicted mask pixel value of the i-th pixel.

M: The true mask pixel values in the pixel matrix.

$\hat{M}$: The predicted mask pixel values in the pixel matrix.

ε: The smoothing constant.

## 4. Experiments and Results

### 4.1. Dataset

**Figure 7:**
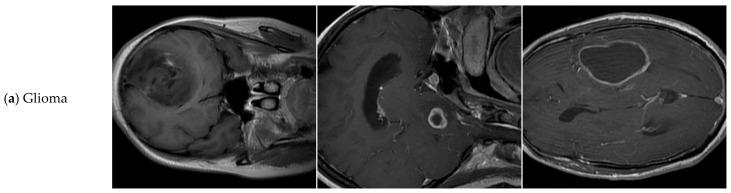

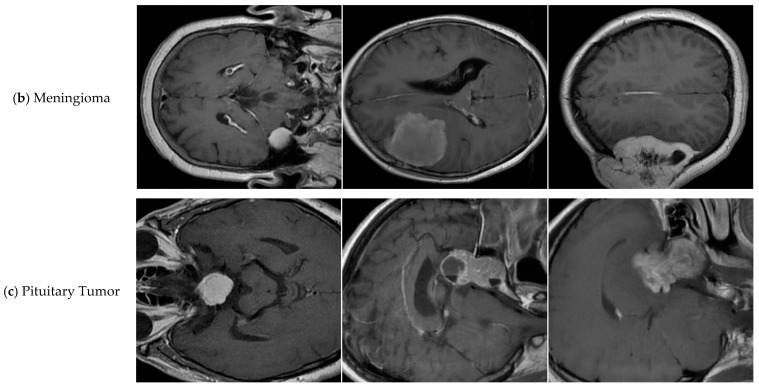
Illustrations of the three types of brain tumors with different shapes and sizes: Glioma, Meningioma, and Pituitary Tumor.

The dataset used in this experiment is named tumor-otak, which is sourced from the Roboflow platform [41]. As shown in Table 1, the dataset comprises 3064 brain tumor MRI images of varying sizes and types, covering the three most common types of brain tumors in clinical practice: Glioma, Meningioma, and Pituitary Tumor (as shown in Figure 7). The dataset images have been divided into training, validation, and test sets in a 7:2:1 ratio, which are used for model training, validation, and performance evaluation, respectively. All images have been annotated in the YOLOv8 format and have undergone uniform preprocessing, including automatic orientation adjustment, removal of EXIF information, resizing to 640 × 640 pixels (with stretching), and grayscale conversion.

### 4.2. Experimental Environment and Training Parameter Settings

The computer environment configuration used for the model in the experiment is shown in Table 2, the main parameters of the model are shown in Table 3, and other parameters are set according to the default configuration of the model.

### 4.3. Evaluation Metrics

The following evaluation metrics are used in this study to measure model performance: Precision, Recall, mAP@0.5(mAP50), Param, Model_Size, and computational complexity, which is represented by Giga Floating-point Operations Per Second (GFLOPs). Precision indicates the proportion of actual brain tumors among all samples detected as positive, reflecting the model’s accuracy. Recall measures the proportion of actual brain tumors detected by the model out of all actual brain tumors. Average Precision (*AP*) assesses the model’s performance on a single class, while mAP@0.5 is the mean of *AP*s across all classes at an Intersection over Union (IoU) threshold of 0.5, used to evaluate the model’s overall performance on multiple classes. In accordance with the evaluation standards of the YOLO series, all experiments in this study adopt mAP as the primary metric, covering object detection performance (mAP@0.5B) and instance segmentation performance (mAP@0.5M). These metrics help to comprehensively evaluate the detection performance, accuracy, and efficiency of the model. The specific calculation formulas are as follows:

(17) $$Precision=\frac{TP}{\mathrm{TP}+FP}$$

(18) $$\mathrm{Recall}=\frac{TP}{\mathrm{TP}+FN}$$

(19) $$\mathrm{AP}=\int_{0}^{1}P_{(r)dr}$$

(20) $$mAP@0.5=\frac{1}{N}\sum_{i=1}^{N}AP_{i}$$

(21) $$GFLOPs=2\cdot H_{out}\cdot W_{out}\cdot C_{in}\cdot C_{out}\cdot K_{h}\cdot K_{w}$$

In Equations (17)–(19), TP (True Positive) represents the number of brain tumors correctly labeled as brain tumors by the model. FP (False Positive) represents the number of non-brain tumors incorrectly labeled as brain tumors by the model. FN (False Negative) represents the number of brain tumors incorrectly labeled as non-brain tumors by the model. $P_{(r)}$ represents the precision at recall rate r. In Equation (20), $H_{out}$ and $W_{out}$ represents the height and width of the output feature map, $C_{in}$ and $C_{out}$ represent the number of input and output channels, and $K_{h}$ and $K_{w}$ represent the height and width of the convolution kernel.

### 4.4. Model Training, Testing, and Results Analysis

**Figure 8:**
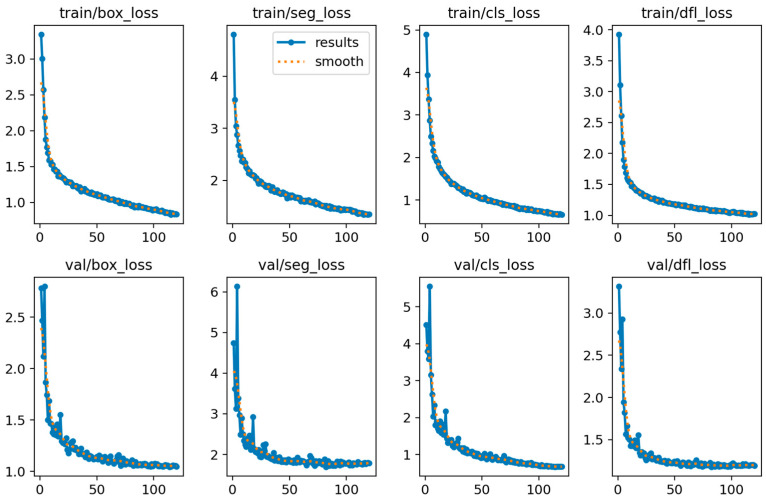
Loss changes in FALS-YOLO on the training and validation sets during the training process.

Figure 8 shows the loss changes in FALS-YOLO on the training and validation sets during the training process. As can be seen from the figure, for both the training and validation sets, the four loss functions (box_loss, seg_loss, cls_loss, and dfl_loss) gradually decrease with the increase in the number of iterations, indicating that the model is continuously optimized and its performance is constantly improved during the training process. Although there are some fluctuations in the loss on the validation set, the overall trend is consistent with that of the training set, which indicates that the model has good generalization ability. These results provide strong support for the effectiveness and stability of the model.

**Figure 9:**
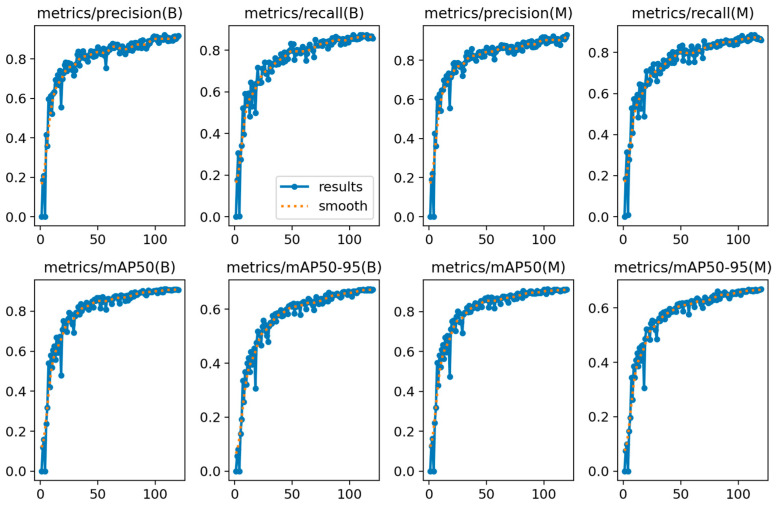
Changes in evaluation metrics (precision, recall, mAP50, and mAP50-95) of FALS-YOLO in the brain tumor detection task (B) and segmentation task (M).

Figure 9 shows the evaluation metrics of FALS-YOLO on the training and validation sets during the training process. In the brain tumor detection task (B) and segmentation task (M), the evaluation metrics (precision, recall, mAP50, and mAP50-95) of the model significantly improve with the increase in the number of iterations and tend to stabilize in the later stages. This indicates that the model is continuously optimized and its performance is constantly improved during the training process. In particular, in the detection task, the model’s mAP50-95 reaches a level close to 0.7, and in the segmentation task, mAP50-95 also reaches a level close to 0.65, which shows that the model has strong generalization ability and robustness in both tasks.

**Figure 10:**
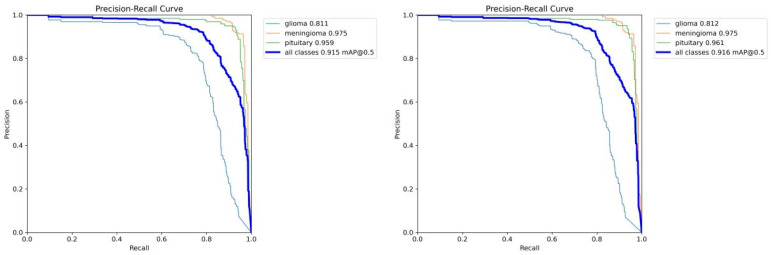
The PR curve changes in FALS-YOLO in the brain tumor detection task (**left**) and segmentation task (**right**) during the training process.

As can be seen from the PR curves in Figure 10, FALS-YOLO performs well in both brain tumor detection and segmentation tasks. For the detection task (left figure), the average precision (mAP@0.5) for all classes reaches 0.915, with mAP values for Glioma, Meningioma, and Pituitary classes being 0.811, 0.975, and 0.959, respectively. This indicates that the model has a high accuracy in identifying these categories. The precision-recall curve shows that precision gradually decreases with the increase in recall, but the overall trend maintains a high-performance level, especially in the high-precision region, demonstrating the model’s effectiveness and robustness. For the segmentation task (right figure), the model also shows excellent performance, with an mAP@0.5 of 0.916 for all classes. The mAP values for each class are similar to those in the detection task, with Glioma at 0.812, Meningioma at 0.975, and Pituitary at 0.961. The precision-recall curve for the segmentation task also shows a similar downward trend, indicating that the model can cover most of the true regions while maintaining high precision. In summary, the model demonstrates strong generalization ability and accuracy in both detection and segmentation tasks, verifying its potential and value in practical applications.

**Figure 11:**
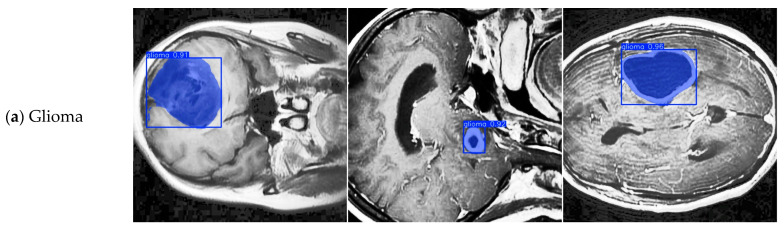

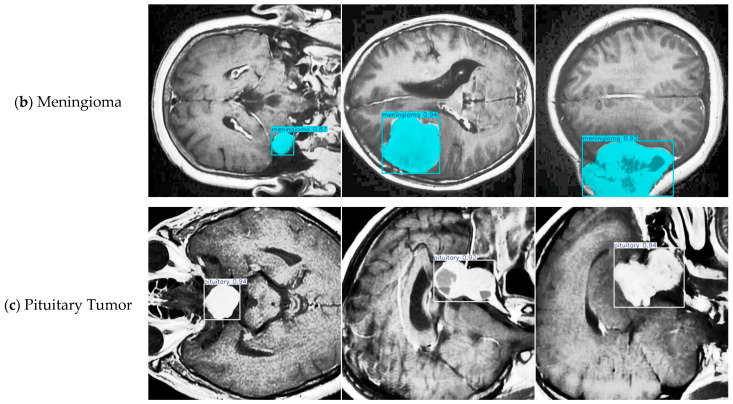
Detection and segmentation results of three types of brain tumors with different shapes and sizes: Glioma, Meningioma, and Pituitary Tumor.

Figure 11 shows the detection and segmentation results of the three types of brain tumors with different shapes and sizes—Glioma, Meningioma, and Pituitary Tumor—in MRI images, as shown in Figure 4. It can be seen from the figure that our model not only accurately locates the position of brain tumors using detection boxes but also clearly segments the contours and sizes of the tumors. At the same time, it identifies the specific types of brain tumors with high confidence. This indicates that our model has excellent comprehensive performance in multi-task learning for detection, segmentation, and classification of different types of brain tumor MRI images. It can provide accurate and reliable image analysis support for the auxiliary diagnosis of brain tumors.

**Figure 12:**
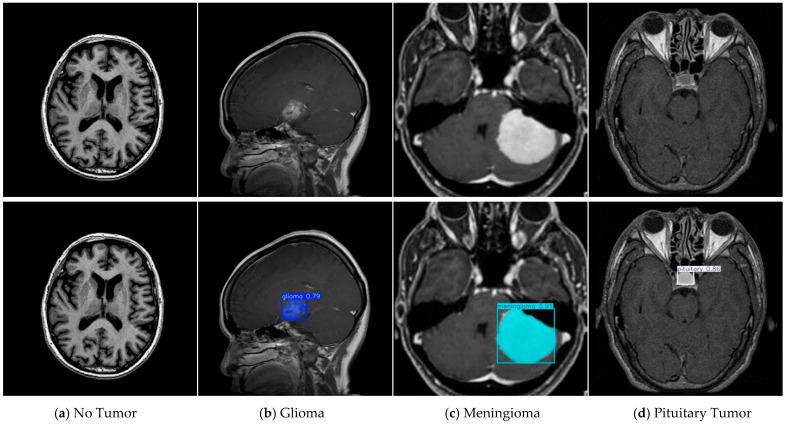
Zero-shot inference results on representative samples, with diagnostic categories sequentially classified as no tumor, glioma, meningioma, and pituitary tumor.

To rigorously assess real-world generalizability, we conducted a zero-shot cross-dataset experiment: FALS-YOLO, trained exclusively on tumor-otak, was directly applied to BRAIN TUMOR [42] (1000 raw MRI images, no preprocessing or augmentation, spanning diverse tumor morphologies, locations, and boundary complexities). Without any fine-tuning, the model reliably localized, segmented, and classified tumors (glioma, meningioma, pituitary tumor), demonstrating superior cross-domain robustness. The experimental results are shown in Figure 12.

### 4.5. Results and Analysis of Ablation Experiments

To verify the effectiveness of the improvements, we conducted a systematic ablation study. We sequentially introduced the FLRDown, AdaSimAM, and LSCSHN modules to assess their impact on model performance. We evaluated the model on the detection task (B) and segmentation task (M) for brain tumors using the tumor-otak test set. The ablation experiment results are shown in Table 4.

In this study, we systematically analyzed the impact of different modules on brain tumor detection (B) and segmentation (M) performance through a series of ablation experiments. The results indicate that the synergistic effect of the modules significantly optimized the model’s precision, recall, mean average precision (mAP), computational complexity, and model size. Specifically, when the FLRDown module was introduced, the Precision (B) for brain tumor detection increased from 0.87 to 0.894, and the mAP@0.5 (B) increased from 0.897 to 0.900, demonstrating the module’s significant advantage in improving detection accuracy. Meanwhile, the number of model parameters decreased to 2,975,529, GFLOPs decreased to 11.5, and the model size decreased to 5.9 M, indicating that the module also played an important role in reducing computational complexity and model size. After further incorporating the LSCSHN module, Precision(B), Recall(B), and mAP@0.5(B) slightly decreased, yet remained at a high level of 0.863, 0.823, and 0.884, respectively, the number of model parameters further decreased to 2,217,318, GFLOPs decreased to 9.6, and the model size decreased to 4.4 M. This shows that the LSCSHN module further optimized model lightweighting while maintaining high detection accuracy. Finally, when the AdaSimAM module was also introduced, the Precision (B) further increased to 0.892, the Recall (B) reached 0.858, and the mAP@0.5 (B) reached the highest value of 0.912. This indicates that the AdaSimAM module has a significant effect on improving detection precision and recall rate while maintaining the model’s lightweight characteristics. At this time, the number of model parameters and GFLOPs remained at 2,217,318 and 9.6, respectively, and the model size was still 4.4M, further proving that the module optimized model performance without adding additional computational burden. In terms of segmentation performance (M), the trends in Precision (M), Recall (M), and mAP@0.5 (M) were similar to those in detection performance. For example, the introduction of the FLRDown module significantly improved Precision (M), the introduction of the LSCSHN plays a crucial role in the lightweighting of the model, while the introduction of the AdaSimAM module improved Precision (M), Recall (M), and mAP@0.5 (M) in segmentation, compensating for the accuracy loss caused by the lightweight design.

Compared with the original YOLOv8n-Seg, the improved model increased Precision by 2.53%, Recall by 1.78%, and mAP@0.5 by 1.67% in brain tumor detection (B), and increased Precision by 2.04%, Recall by 0.58%, and mAP@0.5 by 0.88% in brain tumor segmentation (M). At the same time, the improved model also showed significant optimization in computational complexity and model size. The number of model parameters decreased by 31.95%; GFLOPs decreased by 20.00%; and the model size decreased by 32.31%. In summary, the improved model has achieved significant improvements in both detection and segmentation performance, while significantly reducing computational complexity and model size, indicating that it has higher efficiency and practicality while maintaining high performance.

**Figure 13:**
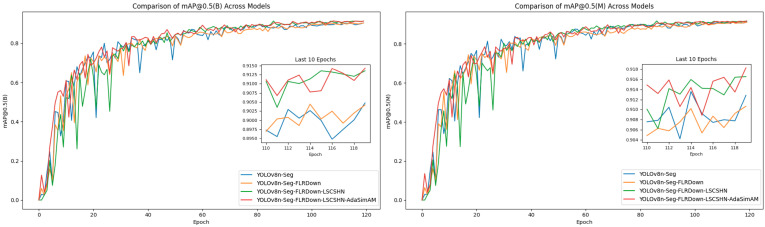
The mAP@0.5 curve changes in the model in the brain tumor detection (B) and segmentation (M) tasks during the training process.

Figure 13 shows the mAP@0.5 curves of the model in the brain tumor detection (B) and segmentation (M) tasks during the training process. The results indicate that all the improved models can quickly enhance performance at the beginning of training and maintain high accuracy and stability in the later stages of training. Compared with the baseline model YOLOv8n-Seg, the detection and segmentation accuracy of the model are improved after the FLRDown module is introduced; the training convergence speed is accelerated and the performance continues to improve after the LSCSHN mechanism is further combined; finally, after the AdaSimAM attention module is integrated, the model achieves the best mAP@0.5 in both object detection and image segmentation tasks, approaching 0.9, showing higher accuracy and better convergence stability. This further demonstrates that the improvement scheme proposed in this study has good practical value in the multi-task scenario of taking into account both detection and segmentation accuracy.

### 4.6. Results and Analysis of Comparative Experiment

To ensure fair and direct comparison under the instance segmentation paradigm, this study benchmarks only lightweight YOLO-family instance segmentation models, including YOLOv5n-Seg, YOLOv8n-Seg, YOLOv9s-Seg, YOLOv10n-Seg, YOLOv11n-Seg, and our proposed FALS-YOLO (Ours).

As reported in Table 5, FALS-YOLO surpasses all counterparts across key metrics, particularly in Param, GFLOPs, and Model_Size. On the detection task, FALS-YOLO achieves a Precision (B) of 0.892, Recall (B) of 0.858, and mAP@0.5 (B) of 0.912—surpassing YOLOv10n-Seg by 1.0, 2.0, and 0.4 percentage points, respectively. On segmentation, it achieves Precision (M) of 0.899, Recall (M) of 0.863, and mAP@0.5 (M) of 0.917, placing it among the top-performing lightweight models. Notably, while YOLOv11n-Seg achieves higher recall values (Recall (B): 0.875, Recall (M): 0.883), FALS-YOLO offers a more balanced precision-recall trade-off and superior efficiency. Crucially, FALS-YOLO accomplishes this with only 2.2 million parameters, 9.6 GFLOPs of computation, and a model size of 4.4 MB—the lowest among all evaluated models. Compared to the heavier YOLOv9s-Seg (8.5M parameters, 17.1 MB), this represents approximately a 74% reduction in parameter count and 74% smaller model size, making it exceptionally suitable for deployment on resource-constrained edge and mobile devices. These results demonstrate that FALS-YOLO achieves an excellent performance–efficiency trade-off, delivering state-of-the-art accuracy for its class while setting a new standard in lightweight design for real-world deployment.

**Figure 14:**
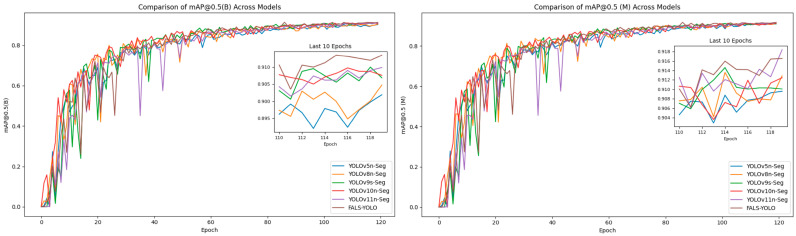
mAP@0.5 evolution of FALS-YOLO, YOLOv5n-Seg, YOLOv8n-Seg, YOLOv9s-Seg, YOLOv10n-Seg, and YOLOv11n-Seg on brain tumor detection (B) and segmentation (M) tasks during training.

Figure 14 illustrates the performance of FALS-YOLO alongside YOLOv5n-Seg and other models on brain tumor detection (B) and segmentation (M) tasks. Across both tasks, FALS-YOLO consistently outperforms its counterparts. During the late training phase of the detection task (B), FALS-YOLO attains markedly higher stability and precision, achieving an mAP@0.5 close to 0.918—substantially exceeding YOLOv5n-Seg, YOLOv8n-Seg, and the remaining baselines. Likewise, in the segmentation task (M), FALS-YOLO reaches the best mAP@0.5, stabilizing around 0.916, underscoring its superiority in accurately delineating tumor regions. Overall, FALS-YOLO delivers superior accuracy and stability for both brain tumor detection and segmentation, establishing itself as a more suitable model for medical image analysis in this domain.

### 4.7. Supplementary Experiment

To comprehensively evaluate the performance enhancement of the AdaSimAM module on the FALS-YOLO model, we conducted a series of comparative experiments. As summarized in Table 6, three configurations were systematically compared: the baseline FALS-YOLO without any attention mechanism, FALS-YOLO integrated with the original SimAM module, and FALS-YOLO enhanced with the proposed AdaSimAM module. This study aims to isolate and quantify the contribution of the AdaSimAM module to the overall detection performance. Experimental results demonstrate that, without introducing any additional computational cost, AdaSimAM significantly outperforms SimAM in both brain tumor detection and segmentation tasks—particularly excelling in recall, thereby effectively reducing the risk of missed diagnoses and validating the effectiveness of the proposed improvement.

**Figure 15:**
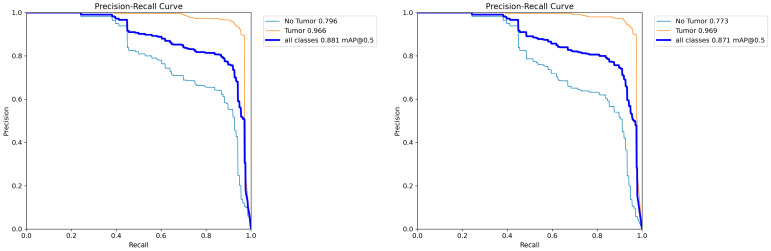
The PR curve changes in FALS-YOLO on the test set for liver tumor detection (**left**) and segmentation (**right**).

**Figure 16:**
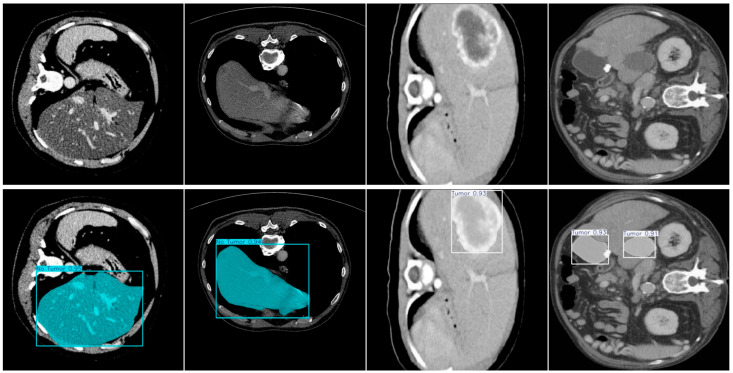
Several samples from the test set of the Project 5C Liver Tumor were successfully detected and segmented into Tumor and No tumor regions.

To enhance data diversity and evaluate the model’s generalization capability, we further evaluated FALS-YOLO on the Project 5C Liver Tumor [43], which consists of 3397 images split into training, validation, and test sets in a 7:2:1 ratio, specifically for liver tumor detection and segmentation. Figure 15 shows that, on the test set of the Project 5C Liver Tumor, FALS-YOLO demonstrates excellent performance in both liver tumor detection and instance segmentation tasks. In the detection task, the model achieves mAP@0.5 scores of 0.796 and 0.966 for the “No Tumor” and “Tumor” classes, respectively, with an overall mAP@0.5 of 0.881, indicating strong lesion identification capability. In the instance segmentation task, segmentation accuracies for the two classes reach 0.773 (No Tumor) and 0.969 (Tumor), with the overall mAP@0.5 remaining at a high level of 0.871. Figure 16 shows several samples from the test set of the Project 5C Liver Tumor, which have been successfully detected and segmented into Tumor and No Tumor regions, with high probability scores assigned. The above results demonstrate that, after training on liver tumor-related data, FALS-YOLO can effectively detect and segment liver tumors, further validating its effectiveness and generalization capability in cross-task scenarios.

### 4.8. Discussion on Brain Tumor Classification Ability

**Figure 17:**
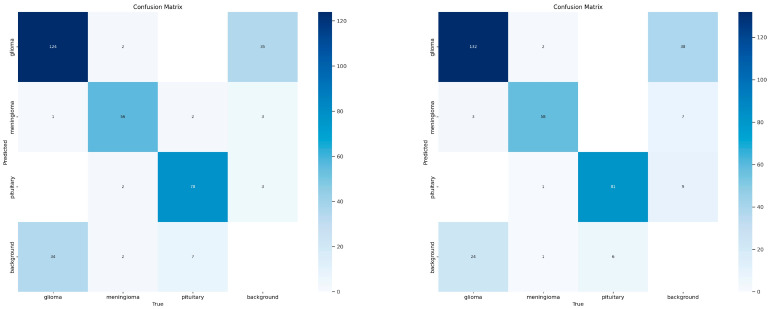
Confusion matrices for the classification of Glioma, Meningioma, and Pituitary Tumor: YOLOv8-Seg (**left**) and FALS-YOLO (**right**).

On the tumor-otak test set, we conducted a comparative analysis of the multi-class brain tumor segmentation accuracy between FALS-YOLO and YOLOv8-Seg results are shown in Figure 17 and Table 7. Confusion-matrix statistics indicate that FALS-YOLO achieves superior overall classification and more reliable lesion identification. Specifically, FALS-YOLO correctly classified Glioma in 132 cases, outperforming YOLOv8-Seg’s 124; for Meningioma and Pituitary tumors, it attained 58 and 81 correct predictions, respectively, slightly surpassing the baseline. Across the three classes, the cumulative correct classifications reached 271, compared with 258 for YOLOv8-Seg. Moreover, FALS-YOLO demonstrated stronger background suppression, misclassifying background regions only 31 times versus 42 for YOLOv8-Seg, indicating greater stability in reducing false positives. In medical imaging, high recall is often more critical than low misclassification; FALS-YOLO’s higher detection rate for key lesions such as Glioma lowers the risk of missed diagnoses, making it more suitable for clinical-assisted diagnostic applications. Overall, FALS-YOLO exhibits superior classification capability and enhanced practical adaptability relative to YOLOv8-Seg.

## 5. Conclusions

In this study, we present FALS-YOLO, a refined YOLOv8-Seg framework tailored for brain tumor instance segmentation. By integrating three key modules, it markedly improves both object detection and instance segmentation on brain tumor MRI images. First, the conventional downsampling layers in the backbone were replaced with our novel FLRDown module, substantially strengthening multi-scale perception and ensuring the reliable recognition and classification of lesions of varying sizes. Second, the neck network was augmented with the adaptive, parameter-free AdaSimAM attention block, which dynamically re-weights feature channels without extra parameters, suppresses noise, and enhances multi-scale fusion, thereby boosting robustness and accuracy. Finally, the original segmentation head was redesigned as the lightweight LSCSHN network, which dramatically reduces computational overhead while preserving high segmentation quality. Comprehensive ablation and comparative experiments were conducted on the tumor-otak dataset. Ablation results reveal that FLRDown primarily elevates accuracy; LSCSHN markedly reduces parameters, FLOPs, and model size, achieving lightweighting; and AdaSimAM, without additional parameters or computations, effectively offsets the performance loss caused by LSCSHN. When the three modules are jointly employed, the model achieves significant gains in both tasks: compared with YOLOv8n-Seg, detection Precision, Recall, and mAP@0.5 improve by 2.53%, 1.78%, and 1.67%, respectively, while segmentation Precision, Recall, and mAP@0.5 improve by 2.04%, 0.58%, and 0.88%. Simultaneously, parameters, GFLOPs, and model size are reduced by 31.95%, 20.00%, and 32.31%, endowing the model with high performance and excellent lightweight characteristics suitable for resource-constrained medical environments. Benchmark comparisons against YOLOv5n-Seg, YOLOv8n-Seg, YOLOv9s-Seg, YOLOv10n-Seg, and YOLOv11n-Seg show that FALS-YOLO consistently delivers superior performance and lightweight properties. For detection (B), it attains 0.892 Precision, 0.858 Recall, and 0.912 mAP@0.5; for segmentation (M), it achieves 0.899 Precision, 0.863 Recall, and 0.917 mAP@0.5, leading all key metrics. Meanwhile, FALS-YOLO has only 2.22 million parameters, 9.6 GFLOPs, and a 4.4 MB model size, all lower than those of other comparative models, demonstrating its outstanding lightweight design. Furthermore, in multi-class tumor classification, FALS-YOLO outperforms YOLOv8-Seg in both classification accuracy and recall rate, exhibiting superior classification capability. We also conducted zero-shot cross-dataset evaluation of FALS-YOLO on BRAIN TUMOR and further examined its liver -tumor detection and segmentation performance on the Project 5C Liver Tumor, collectively demonstrating robust generalizability across organs, imaging protocols, and unseen pathological presentations.

In future research, we will focus on applying FALS-YOLO to brain tumor diagnosis in real-world clinical settings to comprehensively evaluate its operational performance and stability in practical scenarios. Nevertheless, curating large-scale, high-quality medical instance segmentation datasets in YOLO-compatible format remains arduous. We therefore prospectively plan to aggregate multi-institutional, multi-vendor brain tumor cohorts of substantially larger size for continued model refinement, and port FALS-YOLO to the NVIDIA Jetson Nano edge-computing platform to quantify latency, throughput, and energy efficiency under stringent hardware constraints, thereby establishing the technical groundwork for future integration into routine clinical workflows.

## Figures and Tables

**Table 1:** Composition of the tumor-otak dataset.

Tumor-Otak	Total	Glioma	Meningioma	Pituitary Tumor
train	2144	984	502	658
val	612	285	142	185
test	308	159	62	87

**Table 2:** Computer Environment Configuration.

Item	Version
Operating System	64-bit Linux
Programming Language	Python 3.8.19
PyTorch	1.13.1
CUDA	11.7
CPU	Intel(R) Xeon(R) Gold 5218 CPU @ 2.30 GHz
GPU	NVIDIA GeForce RTX 3090 × 1 (24 GB)

**Table 3:** Configuration of the main parameters of the model.

Item	Version
epoch	120
batch	32
workers	4
optimizer	SGD
learning rate	0.001
momentum	0.973
weight decay	0.0005

**Table 4:** Ablation experiment results.

FLRDown	LSCSHN	AdaSimAM	Precision (B)	Recall (B)	mAP@ 0.5(B)	Precision (M)	Recall (M)	mAP@ 0.5(M)	Param	GFLOPs	Model_Size
-	-	-	0.870	0.843	0.897	0.881	0.858	0.909	3,258,649	12.0	6.5 M
√	-	-	0.894	0.823	0.900	0.896	0.84	0.905	2,975,529	11.5	5.9 M
√	√	-	0.863	0.823	0.884	0.877	0.834	0.898	2,217,318	9.6	4.4 M
√	√	√	0.892	0.858	0.912	0.899	0.863	0.917	2,217,318	9.6	4.4 M

**Table 5:** Performance and lightweight comparison of FALS-YOLO versus YOLOv5n-Seg and other models on the detection (B) and segmentation (M) tasks.

Method	Precision (B)	Recall (B)	mAP@ 0.5 (B)	Precision (M)	Recall (M)	mAP@ 0.5 (M)	Param	GFLOPs	Model_Size
**YOLOv5n-Seg**	0.873	0.834	0.900	0.883	0.842	0.913	2,755,945	11.0	5.5 M
**YOLOv8n-Seg**	0.870	0.843	0.897	0.881	0.858	0.909	3,258,649	12.0	6.5 M
**YOLOv9s-Seg**	0.863	0.852	0.895	0.862	0.881	0.906	8,521,017	75.4	17.1 M
**YOLOv10n-Seg**	0.882	0.838	0.908	0.903	0.847	0.915	2,839,449	11.7	5.7 M
**YOLOv11n-Seg**	0.876	0.875	0.907	0.883	0.883	0.913	2,835,153	10.2	5.7 M
**FALS-YOLO** **(Ours)**	0.892	0.858	0.912	0.899	0.863	0.917	2,217,318	9.6	4.4 M

**Table 6:** Model performance with and without the improved AdaSimAM.

SimAM	AdaSimAM	Precision (B)	Recall (B)	mAP@ 0.5 (B)	Precision (M)	Recall (M)	mAP@ 0.5 (M)	Param	GFLOPs	Model_Size
-	-	0.863	0.823	0.884	0.877	0.834	0.898	2,217,318	9.6	4.4 M
√	-	0.887	0.822	0.896	0.895	0.846	0.908	2,217,318	9.6	4.4 M
-	√	0.892	0.858	0.912	0.899	0.863	0.917	2,217,318	9.6	4.4 M

**Table 7:** Integrated confusion-matrix data of FALS-YOLO versus YOLOv8-Seg. In this table, the “Misclassified” column denotes the total number of instances in which a given class was incorrectly assigned to any other class. For the Background category, only the counts of Background being misclassified as another class are reported, since the correct classification of Background itself is usually not of interest.

Class	FALS-YOLO Correct Classification	FALS-YOLO Misclassification	YOLOv8-Seg Correct Classification	YOLOv8-Seg Misclassification
**Glioma**	132	40	124	37
**Meningioma**	58	10	56	6
**Pituitary**	81	10	78	5
**Background**	-	31	-	42

## Data Availability

The public dataset used in this study can be accessed through reference [41,42,43].
